# Crystal Structure of an Integron Gene Cassette-Associated Protein from *Vibrio cholerae* Identifies a Cationic Drug-Binding Module

**DOI:** 10.1371/journal.pone.0016934

**Published:** 2011-03-03

**Authors:** Chandrika N. Deshpande, Stephen J. Harrop, Yan Boucher, Karl A. Hassan, Rosa Di Leo, Xiaohui Xu, Hong Cui, Alexei Savchenko, Changsoo Chang, Maurizio Labbate, Ian T. Paulsen, H. W. Stokes, Paul M. G. Curmi, Bridget C. Mabbutt

**Affiliations:** 1 Department of Chemistry and Biomolecular Sciences, Macquarie University, Sydney, New South Wales, Australia; 2 School of Physics, University of New South Wales, Sydney, New South Wales, Australia; 3 St Vincent's Centre for Applied Medical Research, Sydney, New South Wales, Australia; 4 Department of Civil and Environmental Engineering, Massachusetts Institute of Technology, Cambridge, Massachusetts, United States of America; 5 Banting and Best Department of Medical Research, University of Toronto, Toronto, Ontario, Canada; 6 Institute for the Biotechnology of Infectious Diseases, University of Technology, Sydney, New South Wales, Australia; University of Cambridge, United Kingdom

## Abstract

**Background:**

The direct isolation of integron gene cassettes from cultivated and environmental microbial sources allows an assessment of the impact of the integron/gene cassette system on the emergence of new phenotypes, such as drug resistance or virulence. A structural approach is being exploited to investigate the modularity and function of novel integron gene cassettes.

**Methodology/Principal Findings:**

We report the 1.8 Å crystal structure of **Cass2**, an integron-associated protein derived from an environmental *V. cholerae*. The structure defines a monomeric beta-barrel protein with a fold related to the effector-binding portion of AraC/XylS transcription activators. The closest homologs of **Cass2** are multi-drug binding proteins, such as BmrR. Consistent with this, a binding pocket made up of hydrophobic residues and a single glutamate side chain is evident in **Cass2**, occupied in the crystal form by polyethylene glycol. Fluorescence assays demonstrate that **Cass2** is capable of binding cationic drug compounds with submicromolar affinity. The **Cass2** module possesses a protein interaction surface proximal to its drug-binding cavity with features homologous to those seen in multi-domain transcriptional regulators.

**Conclusions/Significance:**

Genetic analysis identifies **Cass2** to be representative of a larger family of independent effector-binding proteins associated with lateral gene transfer within *Vibrio* and closely-related species. We propose that the **Cass2** family not only has capacity to form functional transcription regulator complexes, but represents possible evolutionary precursors to multi-domain regulators associated with cationic drug compounds.

## Introduction

The *Vibrio* genus is ubiquitous and abundant throughout the aquatic environment. It is clear that lateral gene transfer (LGT) events play a major role in the evolution and adaptation of this organism, with genetic interchange of *Vibrio* genes observed over a wide range of phylogenetic distances [1]. Our analysis of *V. cholerae* and *V. vulnificus* genomes suggests up to 20% of their content to have arisen via this route. The continued emergence of novel pathogenic clones carrying diverse combinations of phenotypic and genotypic properties significantly hampers control of the disease [2]. The emergence of *V. cholerae* O139, one of the two strains responsible for epidemic Asiatic cholera, appears to be a result of LGT from multiple and diverse descendants of the seventh pandemic O1 El Tor strain [2], [3]. Recent studies have indicated that the O1 and O139 associated virulence genes (or their homologues) are also dispersed among environmental strains of *V. cholerae* [4], [5]. LGT and acquisition of virulence genes is then a very likely mechanism for the emergence of pandemic strains of *V. cholerae* from non-pathogenic environmental strains [6], [7], [8], [9]. The mobilization and integration of mobile gene clusters carrying genes for multiple antibiotic resistance, although not directly implicated in the mechanism of pathogenicity, are also thought to significantly influence the epidemiology of cholera [10].

One important mediator of LGT involves mobile gene cassettes clustered in association with integrons [11]. Gene cassettes are captured by integrons via their intrinsic site-specific recombination system [12], [13], [14] and constitute the smallest known mobilisable genetic element [7], [13], [15]. Integrons themselves can be found on mobile elements as well as in the chromosome [7], [15]. While most integron cassette arrays contain relatively small numbers of cassettes, extremely large arrays (numbering 100–200) appear particularly prevalent for *Vibrio* species [8], [16]. Rearrangements and deletions/insertions of large portions of these mobile gene arrays appear to be common events [6], [14], and arrays can display high levels of diversity even in strains that are otherwise closely related. Independent studies continue to show that gene cassettes possess a very high proportion of genetic novelty, whether derived from defined strains [8], [16] or from metagenomic surveys [17], [18].

In this work, we focus on one integron gene cassette (*Vch_cass2*) isolated from a strain of *V. cholerae* resident within a brackish coastal environment in north-eastern USA. Initial sequencing identified the gene cassette to encode a domain with some homology to the AraC superfamily of transcription activators, generally implicated in the regulation of stress response and virulence [19]. These regulators are well characterized to be modular systems, and include the AraC, MarR and MerR protein families [20]. Generally, these are organized with a DNA-binding domain that acts as a positive regulator of transcription fused to an effector domain which provides a binding site for a specific chemical activator molecule [20], [21]. The modularity of these systems provides capacity for complex regulatory networks, which can also incorporate the membrane transporters for extrusion of multiple toxic agents or drugs [22]. In this way, for example, the AraC and MerR multi-domain regulators are organised to be capable of recognizing the same array of toxic compounds extruded by the transporters they themselves transcribe [23].

Our recovery of a gene cassette encoding a single and independent effector-like domain is noteworthy as a likely evolutionary precursor to a transcription regulatory system within *Vibrio spp.* The structural and functional characterisation of this novel integron-associated protein, named here **Cass2**, was thus of immediate interest as a potential drug-binding factor, particularly as the integron/gene cassette system is strongly associated with the emergence of antibiotic and drug resistance [11]. We found the protein structure to be representative of several single-domain homologues, often mobile, within the genomes of related aquatic-dwelling bacterial species. The origin of the gene cassette within an environmental *Vibrio* species points to its potential as a mobile element facilitating the spread of drug resistance and the emergence of novel phenotypes.

## Results

### An Independent Effector-Binding Domain Related to the AraC_E_bind Superfamily

**Figure 1:**
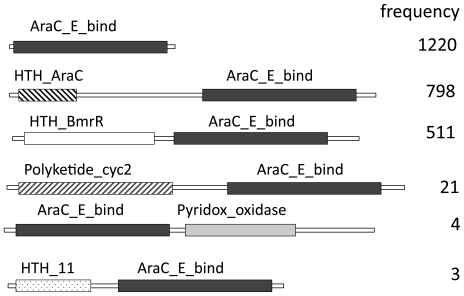
Domain analysis of Cass2 sequence. Six groupings are identified to contain AraC_E_bind (pfam06445) domains in a variety of architectures. The number of sequences found in each grouping (May, 2010) is indicated by frequency.

**Figure 2:**
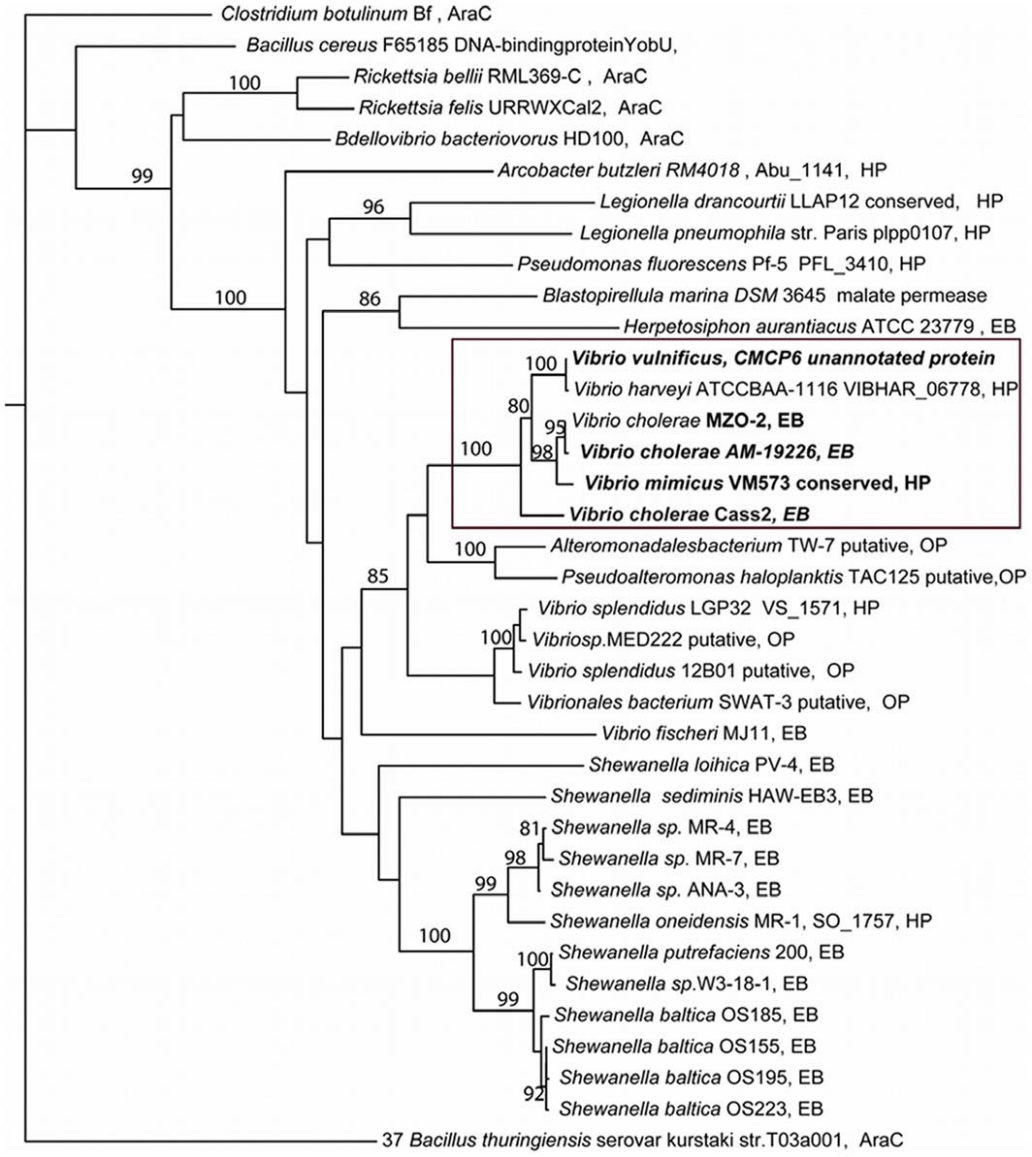
Phylogenetic tree of Cass2 sequence with evolutionary distances. Bootstrap values ≥80% are shown over the internodes for 36 sequence homologues. Protein names are annotated as in the NCBI database and distinguished as: EB, transcription activator, effector binding; AraC, transcription regulator, AraC family; HP, hypothetical protein or OP, orphan protein. Boxed clade contains sequence of interest; its relatives shown in bold are known to be gene cassettes from integron cassette arrays.

**Figure 3:**
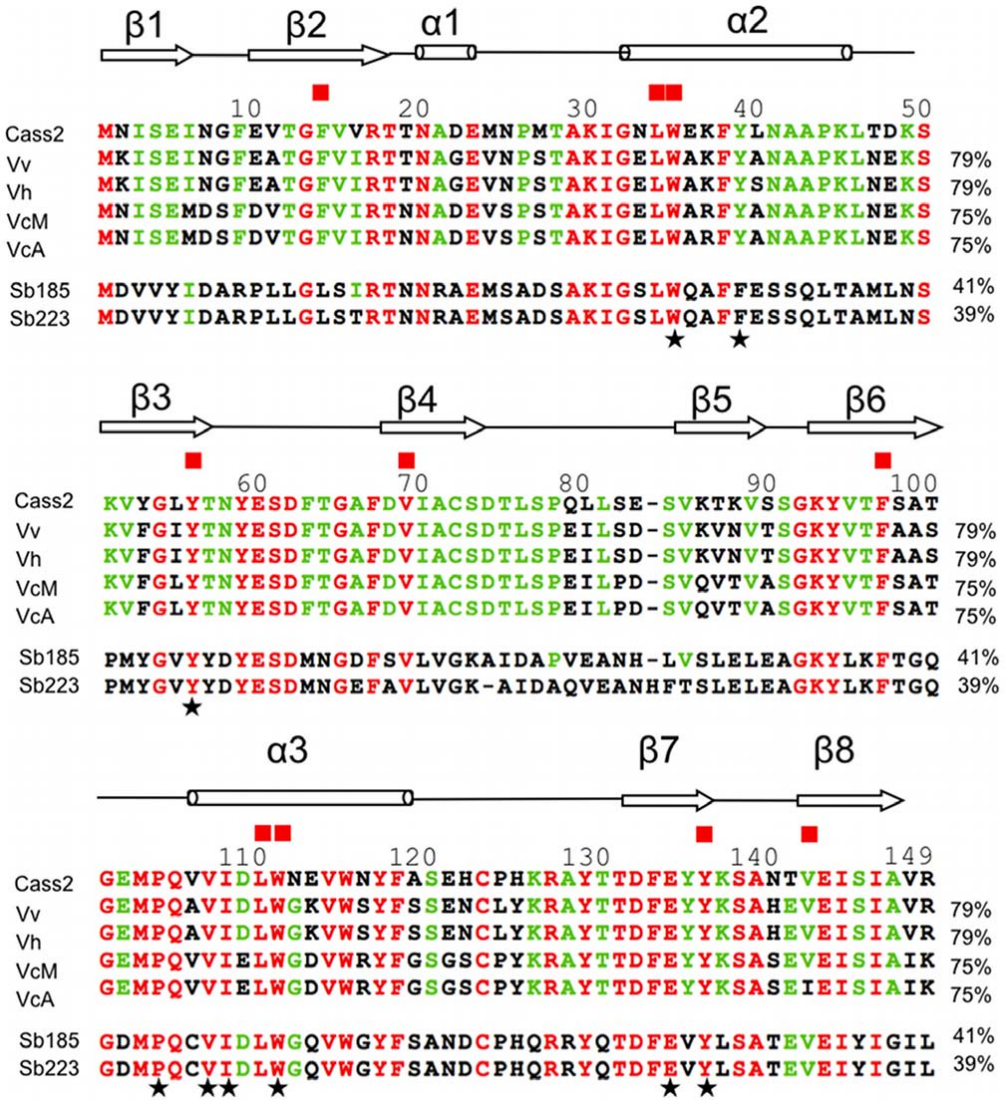
Multiple alignment of Cass2 and sequence homologs. **Cass2** sequence is aligned with representative strains from *Vibrio*: Vv, *Vibrio vulnificus* CMCP6 (AE016795.2); Vh, *Vibrio harveyii* ATCC-BAA-1116 (YP_001448888.1); VcM, *Vibrio cholerae* MZO-2 (ZP_01980402.1); VcA, *Vibrio cholerae* AM-19226 (YP_002176820.1). Representative sequences are also included from the related clade of *Shewanella* homologs: Sb185, *Shewanella baltica* OS185 (YP_0013657); Sb223, *Shewanella baltica* OS223, (YP_0023586). Secondary structure representation is as derived from crystal structure of **Cass2** (this work). Residues completely conserved in all homologs are shaded red, partially conserved residues are green. Active site residues are starred, residues conserved in both subdomains I and II are also indicated (filled square).

The gene cassette named *Vch_cass2* was one of a group of integron gene cassettes isolated from OP4G, an environmental strain of *V. cholerae* derived from a brackish coastal pond in Massachusetts (USA). Partial genomic sequencing has established this strain to have strong sequence identity (>90%) with known pathogenic strains of *V. cholerae* (Boucher, unpublished). The encoded protein sequence, **Cass2**, displays signature motifs that associate it with the superfamily AraC_E_bind (cl01368, sm00871 [24], pfam06445), named for the effector domain of the AraC/XylS transcription activators [21]. Members of this superfamily regulate diverse bacterial functions, including sugar catabolism and responses to stress and virulence [19]. As outlined in Figure 1, several multi-domain protein families incorporate an effector domain of this type (usually C-terminal in position), often in conjunction with a helix-turn-helix DNA-binding domain. This allows transcription activation of cognate promoters to be enabled through the highly conserved DNA-binding domain in response to effector binding [21], [25]. However, in the case of *Vch_cass2*, sequence searches (both gene and protein levels) established it to be representative of an entirely distinct family of independent single-domain proteins, represented by over 1200 homologs across a range of organisms. A phylogenetic analysis of these sequence relatives (Figure 2) places **Cass2** in a distinct clade (75–79% amino acid identity) sourced from a variety of marine-dwelling bacteria. While **Cass2** clearly clusters with homologs from specific *Vibrio spp* (bootstrap value of 100%), a related but distinct clade displaying ∼40% amino acid identity is evident within *Shewanella* genomes. Representative protein sequences for members of these two clades are aligned with that of **Cass2** in Figure 3.

Importantly, like *Vch_cass2*, the genetic context for many of its homologs indicate an association with LGT. Those relatives displaying highest sequence homology are also encoded within gene cassette elements (e.g. *V. cholerae* MZO-2 and AM-19226), while others are found adjacent to transposon features (*V. vulnificus* CMCP6).

### Crystal Structure of Cass2

**Figure 4:**
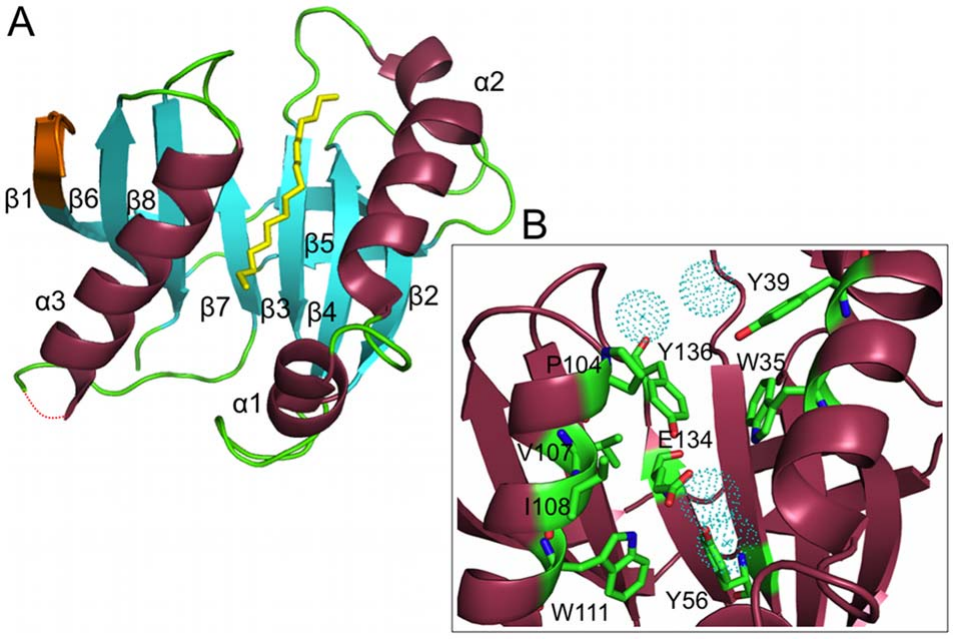
Three-dimensional crystal structure of Cass2 at 1.8 Å. A) Structure coloured by secondary structure elements: strands β1 (residues 1–6), β2 (9–18), β3 (52–57), β4 (67–73), β5 (85–89), β6 (92–100), β7 (131–135) and β8 (141–147) and helices α1 (20–24), α2 (31–45) and α3 (105–118). A loop region of poor density connecting α3 to β7 (residues 120–122) is represented by dotted line. A bound molecule of PEG (yellow) occupies the active site. N-terminal tag residues forming part of strand β1 are shaded orange. B) Depiction of side chains within 4 Å of the PEG molecule are depicted, including surrounding water molecules. Polarity of Glu134 is neutralised by polar groups of Tyr 56, Tyr136 and Trp111 side chains.

Structure determination by x-ray crystallography revealed the protein **Cass2** to be a monomer organised into a barrel-like form comprising an antiparallel β-sheet of eight strands (Figure 4a). Flanking the concave face of the central sheet are two separated helical elements, in which helices α1 (residues 20–24) and α2 (31–45) are aligned to one side, and helix α3 (104–118) to the other. The topology of the domain highlights its pseudo two-fold symmetry, which is based on repeating β-β-α-β-β motifs. Subdomain I (residues 9–91) superimposes over subdomain II (residues 1–8, 92–149) with an rmsd of 1.7 Å (calculated on 37 C_α_ atoms), and directly aligns elements β2, α2, β3 and β4 with β6, α3, β7 and β8, respectively. Despite the low sequence identity of the two subdomains (∼12%), the structural superposition coherently maps side chains Phe14, Leu34, Trp35, Tyr56 and Val69 from subdomain I to those of Phe97, Leu110, Trp111, Tyr136 and Val142 in subdomain II. These recurring side chains stabilise packing of the helices to the β-sheet and form critical elements of the ligand-binding site.

Within both subdomains of **Cass2**, a relatively flexible loop is located C-terminal to the helical portion, i.e. forming connections between α2–β3 (residues 46–50) and α3–β7 (residues 119–130) segments. These loops project from the top and bottom of the sheet, respectively (orientation as depicted in Figure 4). Additional areas of flexibility (as evidenced by elevated B-factors) reside within subdomain I, provided by the loops connecting sheet strands β3–β4 and β4–β5 of the structure.

The central cavity enclosed between the helices of **Cass2** is largely hydrophobic in nature, and aromatic side chains predominate. However, a single acidic group (Glu134, originating from strand β7) is buried deep within this cleft, flanking the pseudo two-fold axis of the protein structure. The polarity of this side chain is stabilised by hydrogen bonds to side chains of Tyr56, Tyr136, and Trp111 (Figure 4B). Between the helical edges of the cavity and directly above the topological switch-point of the sheet (i.e. β3/β7), density is observed corresponding to a polyethylene glycol (PEG) molecule captured during crystallization of **Cass2**. Hydrophobic side chains from helices α2 (Trp35, Tyr39) and α3 (Pro104, Val107, Ile108, Trp111) and the β7/β8 interstrand loop (Tyr136) are within 4 Å of this ligand. Some additional density can be distinguished in our maps belonging to a second (non-definable) ligand, extending further along this same cavity to Trp115.

The sequence alignment for the two distinct clades of **Cass2** relatives from *Vibrio* and *Shewanella* (Figure 3) highlights that conserved sequence segments are distributed throughout the domain, most strongly within structural components making up the central cavity. All of the side chains listed above as interacting with bound PEG, as well as Glu134, are conserved across the **Cass2** sequence family (Tyr39 being conservatively replaced in *Shewanella* strains) (Figure 3). The domain we define here thus provides a common framework for a hydrophobic ligand chemistry.

### Structural Relationship to Effector-Binding Domains

Searches for structural homologues of Cass2 revealed several fold relatives with overlapping biological functions associated with transcription regulation. Close spatial alignment was found to putative transcription regulation protein from *Staphylococcus aureus* (PDB 3LUR), the C-terminal domain of Rob transcription factor from *E. coli* (PDB 1D5Y) [26], the C-terminal drug-binding domain of the multi-drug efflux transporter regulator BmrR from *Bacillus subtilis* (PDB 3D6Z) [27], and the gyrase inhibitory protein GyrI/Sbmc from *E. coli* (PDB 1JYH) [28]. Despite their highly diverse sequences (with only 15–26% identity to Cass2), these three structures overlay well with that of Cass2, with rmsd values of 1.9, 2.1, 2.5 and 2.4 Å, respectively. Some members of the BmrR subfamily, those of the MerR transcription activator systems [20], had already been detected as remote relatives of Cass2 within our initial sequence searches (outlined in Figure 1). The *E. coli* Rob and GyrI domains are also members of the AraC/XylS family of transcription factors; Rob is known to control diverse regulons in prokaryotes [26] and GyrI plays a role in protecting cells against the ribosomally synthesized peptide antibiotic, microcin B17 [28]. Both GyrI and the C-terminal domain of Rob have been speculated to be ligand-binding domains, although the physiological ligands have not been identified.

**Figure 5:**
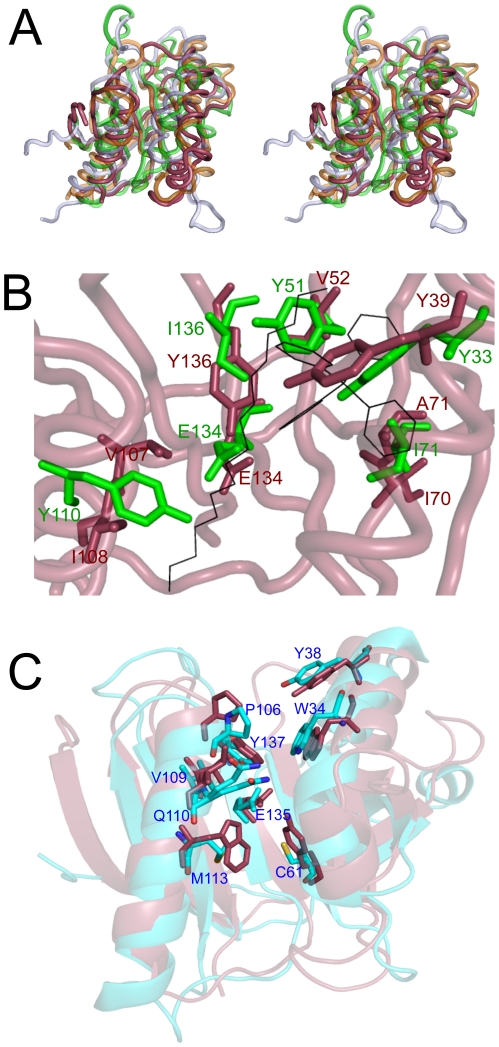
Structural homologs of Cass2. A) Stereo superposition (wall-eyed view) of **Cass2** crystal structure (red), C-terminal domain of Rob transcription factor, E.coli (purple, PDB 1D5Y) [26], gyrase inhibitory protein GyrI, E.coli (orange, PDB 1JYH) [28], C-terminal drug-binding domain of BmrR, B. subtilis (green, PDB 3D6Z) [27]. B) Overlay of active site of drug-bound BmrR (green, PDB 2BOW) on **Cass2** (red) identifies analogous positions of ligand binding residues. Bound TPP and PEG molecules depicted in black. Coordinates of sidechain Tyr33 of BmrR are separately taken from PDB 1R8E [66]. C) Overlay of putative transcription regulation protein from *Staphylococcus aureus* (cyan, PDB 3LUR) on **Cass2** (red) identifies altered chemistry of cavity residues.

Amongst these five structural relatives (overlaid in Figure 5), all display a similar disposition of secondary structure elements, the greatest variation occurring in the region corresponding to helices α2 and α1 of **Cass2**. A glutamate residue is preserved midway across the sheet in all the proteins, stabilised within a hydrophobic environment by surrounding Tyr side chains. The closest structural homolog from *Staphylococcus aureus* (PDB 3LUR), retains many of the hydrophobic side chains of the central cavity, but also possesses a cluster of polar residues (Cys, Gln and Met) not present in **Cass2** (Figure 5C).

For BmrR, known to bind a diverse group of hydrophobic cationic compounds, several crystal structures of its complexes have been determined: those with rhodamine 6G [27], tetraphenylphosphonium (TPP) [29] and berberine [27]. In our structure of **Cass2**, the site occupied by PEG correlates closely with the location of the cationic drug-binding cavity of BmrR [27], [29]. Within the BmrR-TPP complex [29], the phenyl ligand substituents are seen to stack with hydrophobic side chains which include Tyr51 (from strand β3) and Ile71 (strand β4). Nearby, the charged Glu134 residue is stabilized by hydrogen-bonding to the internal tyrosine side chains (Y33, Y68, Y110). Although not all cavity-forming residues of **Cass2** have directly conserved sequence locations in BmrR, a similar binding framework is common to both homologs, as depicted in Figure 5B.

The crystal structure of the **Cass2**-PEG complex displays a markedly distinct conformation in the region C-terminal to helix α2. Brennan's team have proposed that hinge opening of BmrR in the vicinity of helix α2, as well as repositioning of Tyr33 (corresponding to **Cass2** Tyr39), results in the exposure of the central cavity for interaction with the cationic ligand [29]. The loop segment following helix α2 in **Cass2** appears to be relatively flexible in our structure, and it is thus feasible that access to the ligand site in the gene cassette domain might occur by a similar helix-opening mechanism, perhaps coupled in this case with expulsion of the interior side chain Tyr39.

### Ligand Binding Capacity of Cass2

Although the natural ligand of **Cass2** is unknown, it is clear that the domain contains a binding site suitable for hydrophobic/cationic compounds, compatible with that seen in its structural homologs. Tryptophan fluorescence was used to test for interactions of **Cass2** with a set of cationic compounds known to associate with the related bacterial transcription regulators: TPP, benzalkonium chloride, chlorhexidine [30]. The site-specific mutant **(E134Q)Cass2**, designed to neutralise the electrostatic effects of Glu134, was additionally probed in these titrations. **Cass2** contains three tryptophan residues, two of which (Trp35, Trp111) are observed to be in close contact to PEG from helices α2 and α3 within the binding cleft. The third side chain (Trp115 on helix α3) is somewhat more remote along the ligand cavity; it exhibits multiple rotamer forms in the crystal, possibly due to accommodation of other ligand molecules.

**Figure 6:**
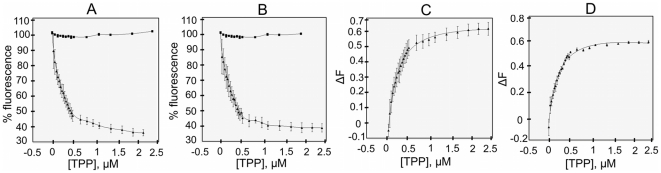
Titration of Cass2 with cationic ligand. Fluorescence quenching is plotted during tetraphenylphosphonium chloride (TPP) binding to **Cass2** (▴) and free tryptophan (▪) for A) wild-type and B) E134Q mutant forms of the protein. Relative fluorescence quenching (ΔF) values were calculated by non-linear regression plot for C) wild-type and D) mutant, leading to determination of K_D_ values (0.2 µM). Fluorescence emission was monitored at 350 nm following excitation at 295 nm (slit widths 10 and 5 nm, respectively).

Initial fluorescence measurements in the presence of excess quantities of all three compounds detected a blue shift (5 nm) from the emission maximum of **Cass2** in its apo form (349 nm). This is consistent with loss of solvent exposure of the Trp residues, such as might occur as the cavity closes upon ligand binding. For all three compounds at sub-micromolar concentrations, significant quenching (up to 60%) of the intrinsic fluorescence emission of **Cass2** was observed in a concentration-dependent manner, as illustrated for the titration with TPP in Figure 6. All interpolated K_D_ values were determined to be in the sub-micromolar range (Table 1). The monovalent compound benzalkonium chloride, smallest of the three compounds tested, displayed the strongest binding (K_D_ = 0.1 µM). The binding affinity determined for TPP (K_D_ = 0.2 µM) indicates a tighter interaction with **Cass2** than has been reported for the fold relative BmrR [27].

**Table 1:** Ligand binding affinities (K_D_ (µM)) of Cass2 and BmrR for cationic compounds.

	Cass2^a^		BmrR	
	wt	E134Q	wt	E134Q
monovalent:				
benzalkonium chloride	0.10±0.50	0.30±0.06	-	-
tetraphenylphosphonium (TPP)	0.20±0.08	0.20±0.13	74.0±20.5^b^;	62.60±3.30
			100^c^	
divalent:				
chlorhexidine	0.20±0.05	0.10±0.02	-	-

a tryptophan fluorescence quenching experiments (this work), 24°C, pH 7.5.

b from isothermal titration calorimetry binding assays [27].

c from equilibrium dialysis methods [23], [69].

Mutation of the central glutamate sidechain of **Cass2** had little effect on its strength of binding to the monovalent compounds tested (TPP, benzalkonium chloride). For the divalent compound chlorhexidine, the affinity for the E134Q mutant appears to have been somewhat enhanced (K_D_ = 0.10 µM). The electrostatic role of this glutamate thus appears to be tempered in the case of **Cass2**, presumably due to the large number of hydrophobic contacts within the internal binding cavity.

Our results are consistent with the earlier binding studies of BmrR to three cationic compounds (Table 1) and crystal structures obtained for the resulting complexes [27]. Substitution of the central glutamate reside of BmrR (alanine and glutamine variants) resulted in unpredicatable binding affinities for TPP, berberine and rhodamine 6G. This led the Brennan group to propose that the overriding enthalpic contributors to binding affinity are the Van der Waals and stacking interactions between protein and drug compound, rather than charge-charge interactions [27]. This is consistent with our observation of little alteration of tight binding of TPP to **Cass2** with loss of the glutamate charge.

Given we can demonstrate that **Cass2** successfully binds the same cationic compounds known to associate with transcriptional regulators, minimal inhibitory concentration (MIC) assays were undertaken to determine if the *Vch_cass2* gene could directly confer resistance to *Vibrio* cells growing on media containing these compounds. Laboratory strains *Vch_cass2+* and *Vch_cass2* were prepared, but in the presence of all compounds, no difference in cell growth was observed for the two strains. The inability of *Vch_cass2+* gene to directly confer resistance to cationic compounds points to the need for protein factors in addition to the effector domain to be present for effective regulation of their cellular metabolism.

### A Conserved Protein-Binding Interface

**Figure 7:**
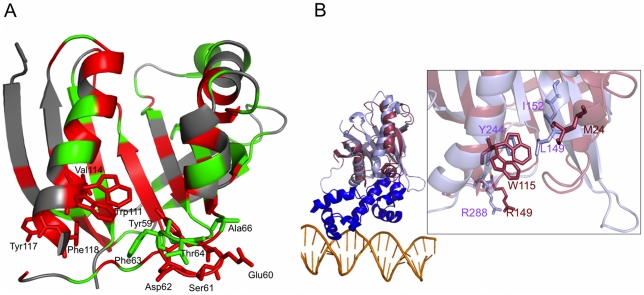
Potential protein-binding interface in Cass2. A) Ribbon structure of **Cass2** coloured according to sequence conservation across the *Vibrio* and *Shewanella* clades (red, fully conserved; green, homologous; see Figure 3). Conserved residues cluster in the PEG-binding cavity and a separate putative protein-binding surface. B) Features of the protein-binding surface in **Cass2** (red) overlay with those of the interface between effector-binding (purple) and DNA-binding (blue) domains of the two-domain Rob protein (PDB 1D5Y) [26]. Inset shows magnified view of domain interface of Rob and analogous key residues in **Cass2**.

Two sequence segments of the **Cass2** sequence family not directly associated with the ligand-binding cleft stand out as strongly conserved. One encompasses the sequence motif -YESD- located from Tyr59 within the β3/β4 loop. When mapped onto the three-dimensional fold of **Cass2**, these side chains, in addition to residues Phe63, Thr64 and Ala66, cluster along a projected surface feature well to the “base” of the binding cleft (depicted in Figure 7A). An additional conserved segment, -VWxYF- (from Val114 in **Cass2**), is the origin of exposed Trp and Phe side chains which elongate the same surface. The entire region is relatively flexible in the crystal structure, with high B-factors observed for the loop residues.

A possible role for this surface becomes evident when, for instance, the structure of **Cass2** is overlaid with that of the two-domain Rob transcription factor [26]. This highlights a remarkable preservation of molecular properties of this surface in both systems (Figure 7B). In the Rob protein, the site clearly forms the interface between the effector-binding (C-terminal) and DNA-binding (N-terminal) domains. Despite being a single module, **Cass2** retains some of the hydrophobic features of the interface, as well as possessing protruding charged side chains, including Arg149 (as its C-terminal residue). In the Rob structure, the analogous side chain at this location (Arg288) participates in an electrostatic interaction across to the neighbouring DNA-binding domain. Thus, **Cass2** gives every appearance of being suitably organised for interaction with a protein partner with features common to the helix-turn-helix domains utilised by its sequence relatives.

It should be noted that the organization of both **Cass2** and Rob differ completely from the situation found in the BmrR fold homolog, the interdomain interface of which is located on the opposite side of the effector-binding module [31]. The BmrR interaction interface entails the packing of the DNA-binding domain of each monomer against the drug-binding domain of its dimerisation partner [31]. Amongst the structural elements necessary for stabilizing this interaction, a 10-residue loop from the drug-binding domain intercalates helices α3′ and α4′ of the DNA-binding domain. The corresponding loop in **Cass2**, connecting strands β7 and β8, is relatively short (136–140) and unlikely to participate in a similar interaction. The absence of a linker helix in **Cass2**, oriented on the same side as the domain interface and essential for dimerisation in BmrR, further rules out this region as a putative protein-binding interface.

## Discussion

Our experimental evidence establishes that the gene cassette *Vch_cass2* encodes a single and independent binding domain for cationic compounds. The structure (and sequence) of its protein product **Cass2** readily confirms its homology to effector-binding domains associated with the AraC/XylS and MerR family of transcription regulators. These well-characterized factors are mediators of bacterial antibiotic and multi-drug resistance through their ability to both recognise effector molecules and to regulate transcription of the appropriate efflux system [21], [25], [30]. Although these multi-domain proteins usually possess similar DNA-binding domains, it is through variation of the effector-binding domain that response and binding is adapted to a range of ligand types.

The crystal structure of **Cass2** depicts PEG in a binding site organised with features reminiscent of those of the effector modules of bacterial regulators [27], [29]. Our fluorescence assays confirmed **Cass2** to be particularly well adapted for tightly binding the cationic drugs which serve as ligands to the AraC/MerR family. Hydrophobic forces appear to predominate within the binding interactions, and (unlike BmrR) the **Cass2** domain is capable of binding monovalent and bivalent ligands. Within the structural framework of **Cass2**, a distinct loop feature extending from helix α2 edging the central sheet (residues 41–46) is proposed to undergo structural rearrangement so as to facilitate ligand entry.

Significant sequence homologies are found between **Cass2** and genes from a group of phylogenetically-related *Vibrio* and *Shewanella* species. The crystal structure presented here therefore defines the paradigm fold for a new family of effector-binding proteins prevalent within these marine-dwelling species. Sequence variation between the two related groups of proteins is restricted to the putative hinge region (C-terminus of helix α2) as well as strand β4. Thus a slightly altered ligand accessibility may have evolved for the distinct clades outlined here.

The association of the *Vch_cass2* gene with mobile DNA elements, also notably evident for its group of related homologs, emphasises the mechanism by which these binding modules can be laterally transferred between species. While the presence of a DNA-binding partner appears necessary for transcription regulation, we cannot rule out the possibility that the biological function of **Cass2** itself may be to provide a self-contained low-level multidrug resistance system, capable of sequestering drugs and preventing them from reaching further intracellular targets. The role of cationic drugs in treatment of cholera and inhibition of cholera toxin-internalization has been previously reported [32], [33], [34]. The depiction in this work of a novel effector domain capable of binding cationic compounds is therefore of immediate interest, given that these are encoded within the mobile integron gene cassette system.

We have, however, noted surface features in the **Cass2** structure consistent with a protein interaction site adjacent to the active-site cavity. We propose this to comprise a potential site for interaction of the effector-binding module with a specific DNA-binding domain, so as to mimic the organisation of the multi-domain transcription regulators. This is congruent with the more general observation that two interacting prokaryotic proteins, not necessarily encoded by neighbouring genes, may be found fused as a single chain homolog in another organism [35], [36], [37]. Such component proteins might be engaged in either direct physical interaction or an indirect functional association [35]. Sequence searches were conducted to locate any likely companion module(s) for **Cass2** in *V. cholerae*; no sequence homolog of the single-domain protein MarA (from *E. coli*) [38] was found amongst gene cassettes from the same environmental isolate as **Cass2**. However, wider sequence searches across published *Vibrio* genomes do reveal the existence of single-domain homologs (ZP_01062623.1; ZP_01976746.1) containing the helix-turn-helix motifs present in both MarA and Rob relatives.

The overall structure of the **Cass2** protein and its relationship to other members of the AraC/XylS and MerR family reinforces the notion that gene cassettes within integron arrays generally move and rearrange independently of one other. Given that many cassettes encode single small domain proteins, loss of intervening *attC* site sequences may lead to permanent fusion of gene cassettes so as to instead encode a multi-domain polypeptide that confers advantage. Our recovery of an independent single domain with effector-binding capacities is significant as a possible evolutionary precursor to the multi-domain transcription regulators, of which the AraC and MerR families are examples.

Evidence for fusion events in the evolution of MerR regulators has previously been outlined [18], [31]. For example, the *tipA* gene of *S. lividans* encodes single and two domain gene products. The full-length gene product (TipAL) comprises an N-terminal helix-turn-helix domain which auto-regulates the *tipA* gene in conjunction with a thiostrepton-binding domain. In vast molar excess, however, a shorter in-frame translational product (TipAS) comprising solely the drug-binding domain is independently transcribed [39], [40], [41]. Thus new types of transcriptional regulators are likely to evolve via gene fusion events incorporating different effector-binding domains coupled to DNA-processing modules. The depiction in this work of a novel effector domain encoded within an integron gene cassette suggests that integrons play an important role in this evolution of complex multi-domain proteins.

## Materials and Methods

### Gene Isolation

Strain OP4G of *V. cholerae* was isolated from a brackish coastal pond (Oyster Pond, Falmouth, MA, USA) as follows. Several water samples (1 ml) were spread directly agar containing on thiosulfate/citrate/bile salts/sucrose (TCBS; commonly used to isolate members of genus *Vibrio*) [42] and incubated overnight at 37°C. Isolated colonies of a yellow colour (i.e. sucrose positive) [43] were picked and re-streaked on tryptic soy broth media. After further overnight incubation, isolated colonies were picked and re-streaked on TCBS media and again incubated overnight. This procedure was repeated twice to ensure pure cultures of the isolates, on which cassette-PCR [44] was performed to isolate integron gene cassettes, including *Vch_cass2*.

### Protein Preparation

**Cass2** was produced recombinantly in *Escherichia coli* strain BL21-CodonPlus (DE3)-RIPL (Stratagene) with an N-terminal affinity tag (MGSSH_6_SSGRENLYFQG-**Cass2**) using the plasmid p15TV-L. **Cass2** was derivatized with selenomethionine (SeMet), as provided within the M9 SeMet media kit (Medicilon, Shanghai) supplemented with antibiotics (ampicillin (100 µg/ml), chloramphenicol (25 µg/ml)). Cells were grown at 37°C until OD_600_ 1.2 and induced with 1 mM IPTG (Medicilon, Shanghai) prior to overnight growth at 25°C. Harvested cells (from 1 l culture) were frozen in Buffer A (50 mM HEPES buffer (pH 7.5), 500 mM sodium chloride, 5 mM imidazole, 5% glycerol) and sonicated in the presence of protease inhibitors (phenylmethylsulphonyl fluoride (0.5 mM) and benzamidine (1 mM).

Following storage (80°C), the soluble cell fraction was loaded onto Ni-nitroloacetic affinity media (Qiagen) washed with Buffer A and eluted with Buffer A containing 250 mM imidazole. After addition of ethylenediamine tetraacetic acid (EDTA, 1 mM), purified **Cass2** was dialysed into Buffer B (10 mM HEPES buffer (pH 7.5), 500 mM sodium chloride) and concentrated to ∼20 mg/ml for crystallization. The reducing reagent tris-(2-carboxyethyl)-phosphine (0.5 mM) was added to all purification buffers.

**(E134Q)Cass2** was prepared using a commercial kit (Quikchange II, Stratagene). The recombinant protein was prepared with *E. coli* BL21 (DE3) Rosetta cells (Merck) in Luria Bertani (LB) medium at 37°C. Following induction (0.2 mM IPTG) and growth at 20°C for 5 h, cells were recovered and the mutant protein isolated from the soluble fraction by batch affinity chromatography (HisTrap, GE Healthcare). Protein buffers were as above.

### Crystallization and Structure Determination

Using sitting-drop format, crystals of **Cass2** were grown to diffraction quality in 0.1 M citric acid (pH 3.50), 25% (w/v) PEG-3350. The crystals (P3_2_21 space group; a = 59.38 Å, b = 59.38 Å, c = 95.76 Å) were cryo-protected by immersion in paratone-N (Hampton Research) prior to flash freezing. Diffraction data was collected at 100 K using synchrotron radiation at the selenium K absorption edge (beamline 19-ID, APS, Argonne National Laboratory).

Diffraction data to 1.8 Å was processed using MOSFLM [45], SCALA [46] and CCP4 software [47]. The structure was solved by SAD using modules of the Phenix suite [48], with anomalous scattering substructure searches and density modification from the AutoSol wizard [49] identifying five Se sites. A preliminary model (88 residues, overall model-map correlation of 0.56) was built and visualized in Coot [50] and monitored throughout refinement (ADIT server) [51]. AutoBuild [52] was used for iterative model building, and the resulting model subjected to 20 macro-cycles of combined TLS, occupancy, coordinate and individual ADP refinement in phenix.refine [53]. An elongated electron density clearly visible in the Fourier difference map during the last refinement cycles was modelled using coordinates for polyethylene glycol (PEG 4000) from the HIC-Up database [54]. Data and refinement parameters are summarized in Table 2.

**Table 2:** Selected crystallographic statistics for Cass2 structure determination.

**Data collection**	
Resolution (Å) (outer shell)	1.8 (1.90 - 1.80)
Unique reflections	18598
Completeness (%) (outer shell)	99.6 (99.7)
I/s(I)>(outer shell)	21 (3.6)
Multiplicity	9.9
R_merge_ a (outer shell)	0.062 (0.561)
Anomalous completeness (outer shell)	99.7 (99.8)
Anomalous multiplicity (outer shell)	4.8 (4.6)
**SAD Phasing statistics**	
Number of SeMet	4
Extent of anomalous signal (Å)^b^	2.4
Refined sites	11
Figure of merit^c^	
acentric, centric, overall	0.419, 0.133, 0.384
**Refinement statistics**	
Solvent content, VS (%)	50.82
R_cryst_/R_free_	0.185/0.227
Reflections in R_cryst_/R_free_	34832/1782
Resolution range (Å)	35.04-1.8
Mean B-factor (Å^2^)	27.22
r.m.s.d. bond lengths (Å)^c^, bond angles (°)^d^	0.008, 1.2
Ramachandran plot^e^	
favoured (%),allowed (%),outliers	96.8, 99.4, 1

a ΣΣ_i_|I_h_−I_hi_|/ΣΣ_i_I_h_, where I_h_ is the mean intensity of reflection h,

b According to AutoSol wizard in Phenix [49],

c According to Phaser [67] in Phenix [48] with resolution 47.88 – 1.80,

d From ADIT Validation server [51],

e From Molprobity [68].

The structure of **Cass2** reveals one chain per asymmetric unit, with electron density visible for 153 residues, including 7 residues of the affinity tag. No density was observed for residues 120–122 (Ser-Glu-His). Residues SeMet1 (strand β1), SeMet24 (helix α1) and Trp115 (helix α2) showed alternative conformations, suggestive of increased mobility within these portions of the molecule. The Ramachandran plot shows >96% of residues in most favoured regions; one outlier (Ser61; average B-factor = 50.1) occurs within an elongated loop (residues 58–66) connecting strands β3 and β4 of the central β-sheet.

### Sequence and Structure Analysis

Sequence homology searches of the non-redundant database (as at Nov, 2009) were performed using PSI-BLAST with a set threshold E-value <10^−10^ and iterated until convergence (11 rounds) [55]. A TBLASTn search was also performed against the translated nucleotide sequence database of the *Vibrio* genus. The retrieved amino acid sequences (248 in total) were subjected to a phylogenetic analysis using a suite of programs within the Mobyle web interface [56]. Multiple sequence alignments were generated using ClustalW [57] and edited using Bioedit [58] to remove gaps. The Phylip package [59] within the Mobyle portal was used to generate a distance matrix tree using Protdist and Neighbor. The confidence of nodes in amino acid analyses was estimated by 1,000 bootstrap replicates generated using SEQBOOT and compiled in a consensus tree with CONSENSE. The resulting tree was viewed with the Drawgram application. CD-Search and CDART tools of NCBI [60] were used to identify related sequence families of **Cass2** and to locate homologs within other domain organizations (as at May, 2010). DALI [61] and PDBeFold (previously SSM) [62] servers were used to identify structural homologs of the crystal structure, as was the SCOP database [63].

### Binding Assays by Intrinsic Tryptophan Quenching

Fluorescence assays were used to detect binding of compounds to **Cass2** and related mutants. Concentrated ligand solutions in Buffer B were titrated into a 400 µl sample of protein (180 nM in Buffer B) and Trp fluorescence monitored. As a control, each compound was also titrated into a 1.3 µM sample of tryptophan (99% purity) in Buffer B, a concentration selected as yielding similar fluorescence to the initial **Cass2** sample prior to titration.

Fluorescence intensities were recorded at 22°C with a PerkinElmer LS55 fluorescence spectrophotometer using a 1 cm×0.2 cm quartz cell. When subjected to an excitation wavelength of 295 nm, **Cass2** displayed maximum emission at 349±1 nm (*apo* form) and 344±1 nm (fully bound). Thus, fluorescence quenching was monitored by recording emission at 350 nm for all samples following excitation at 295 nm (slit widths 10 and 5 nm, respectively) with an integration time of 5 s. All readings were corrected for buffer background emission and sample dilution. Inner-filter effects were measured by titrating each compound into a 1.3 µM sample of tryptophan in Buffer B and the relative fluorescence quenching (ΔF) corrected as follows [64]:

ΔF = (*F*_0_−*F*_C_ (*F*_W0_/*F*_WC_))/*F*_0_ where *F*_0_ = fluorescence intensity of protein sample, *F*_C_ = fluorescence intensity of protein with added compound, *F*_W0_ = fluorescence intensity of free tryptophan solution, *F*_WC_ = fluorescence of tryptophan solution with added compound.

Standard deviation was calculated for the individual ΔF values from three independent experiments. For the determination of dissociation constants (*K*_D_) for the interactions, ΔF was plotted against compound concentration and fitted to the following equation by non-linear regression using Kaleidagraph (Synergy software):

ΔF = ((ΔF_b_−ΔF_f_) [**Cass2**])/(*K_D_*+[**Cass2**]))+ΔF_f_ where ΔF is the relative fluorescence quenching, ΔF_b_ is the maximum relative fluorescence quenching (ligand-saturated **Cass2**); ΔF_f_ is the relative fluorescence quenching of unbound **Cass2**.

### Inhibition Assay

Plasmid pJAK16+*Vch_cass2* was prepared and conjugated into *Vibrio* sp. DAT722 [16] to create strain *Vch_cass2+*. Minimal inhibitory concentration (MIC) assays were conducted with the cationic agents in 96-well plate format using a broth micro-dilution technique [65]. *Vch_cass2+* and *Vch_cass2−* (control strain: *Vibrio* sp. DAT722+pJAK16 plasmid without *Vch_cass2* gene) were grown overnight (37°C) in LB/salt medium. Subcultures (1/100, 1/20 dilutions) were grown at 37°C until OD_600_ value 0.6. Wells were inoculated with 10 µl subculture following further dilution (1/100 in LB/salt medium), and growth monitored by recording OD_595_ after 16 h.

### PDB Accession Number

Coordinates and structure factors for **Cass2** are deposited as PDB file 3GK6.
